# Prospective pathway signaling and prognostic values of MicroRNA-9 in ovarian cancer based on gene expression omnibus (GEO): a bioinformatics analysis

**DOI:** 10.1186/s13048-021-00779-z

**Published:** 2021-02-09

**Authors:** Li Zuo, Xiaoli Li, Yue Tan, Hailong Zhu, Mi Xiao

**Affiliations:** Department of Oncology, Fudan University Shanghai Cancer Center, Minhang Branch, 106 Ruili road, Minhang district, Shanghai, 200240 China

**Keywords:** Ovarian cancer, miR-9, Differentially expressed genes, Functional factors

## Abstract

**Objective:**

MicroRNAs (miRNAs) play a vital role in the development of ovarian cancer (OC). The aim of this study to investigate the prognostic value and potential signaling pathways of hsa-miR-9-5p (miR-9) in OC through literature review and bioinformatics methods.

**Methods:**

The expression of miR-9 in OC was assessed using the public datasets from the Gene Expression Omnibus (GEO) database. And a literature review was also performed to investigate the correlation between miR-9 expression and the OC prognosis. Two mRNA datasets (GSE18520 and GSE36668) of OC tissues and normal ovarian tissues (NOTs) were downloaded from GEO to identify the differentially expressed genes (DEGs). The target genes of hsa-miR-9-5p (TG-miR-9-5p) were predicted using miRWALK3.0 and TargetScan. Then the gene overlaps between DEGs in OC and the predicted TG-miR-9-5p were confirmed using a Venn diagram. After that, overlapping genes were subjected to Gene Ontology (GO) enrichment analysis and Kyoto Encyclopedia of Genes and Genomes (KEGG) pathway analysis. Finally, a protein-protein interaction (PPI) network was constructed using STRING and Cytoscape, and the impact of hub genes on OC prognosis was analyzed.

**Results:**

It was found that OC patients with miR-9 low expression had poor prognosis. A total of 107 DEGs related to both OC and miR-9 were identified. Dozens of DEGs were enriched in developmental process, extracellular matrix structural constituent, cell junction, axon guidance. In the PPI network analysis, 5 of the top 10 hub genes was significantly associated with decreased overall survival of OC patients, namely FBN1 (HR = 1.64, *P* < 0.05), PRRX1 (HR = 1.76, *P* < 0.05), SMC2 (HR = 1.22, *P* < 0.05), SMC4 (HR = 1.31, *P* < 0.05), and VCAN (HR = 1.48, *P* < 0.05).

**Conclusion:**

Low expression of miR-9 indicates poor prognosis of OC patients. MiR-9 plays a crucial role in the biological process of OC by binding to target genes, thus affecting the prognosis of patients.

## Introduction

Ovarian cancer (OC) is one of the most common gynecological malignancies, which poses a serious threat to women’s life and health. OC is clinically characterized by insidious- and late-onset, high grade of malignancy, high metastasis rate, and poor prognosis. Its incidence is increasing yearly, and its mortality rate ranks first compared with other female genital tract malignancies, such as endometrial cancer, cervical cancer and vulvar cancer [1, 2]. The American Cancer Society (ACS) has estimated that there will be approximately 22,530 new cases of OC and 13,980 deaths from OC in the United States in 2019 [2]. Because OC is anatomically located deep within the pelvis, the early lesions have no obvious signs and specific diagnostic methods. Therefore, about 59% of patients have progressed to an advanced stage when they seek medical treatment due to abdominal distension, ascites, and abdominal pain [3]. The 5-year survival rate of patients with early OC can be greater than 75%, while that of patients with advanced OC is only 29% [2]. With the continuous improvement of diagnosis and treatment, the 5-year survival rate of OC patients has increased from 36% in 1975 to 47% in 2014 [4]. However, more than 50% of patients with advanced OC relapse within the first 5 years after treatment and develop cross-resistance to standard chemotherapy and other functionally and structurally unrelated chemotherapeutic agents [5].

Because of the low survival rate and high recurrence rate, it is of great significance to find new targets related to the prognosis of OC, and explore the specific mechanism, so as to perform personalized treatment and to improve clinical efficacy and quality of life of OC patients. Besides,, new targets will also have a significant impact on the detection of cancer recurrence and the guide to rehabilitation.MicroRNAs (miRNAs) are a class of highly conserved endogenous non-coding small RNAs composed of 18–22 nucleotides [6, 7], which play a role in gene regulation in eukaryotes. MiRNAs, as small RNAs that control post-transcriptional gene expression, regulate about 30% of genes in the human genome. One miRNA can regulate the expression of various genes, while one gene can also be regulated by various miRNAs [8]. MiRNAs play a crucial role in cell proliferation, migration, differentiation and apoptosis [9, 10]. It has been reported that miRNAs are also involved in the development of a variety of diseases, such as cancers [11] and cardiovascular diseases [12], and are used as targets for clinical diagnosis and treatment. Increasing studies have shown that aberrant expression of hsa-miR-9-5p (miR-9) is involved in biological processes in various cancers. For example, miR-9 can inhibit the proliferation, migration, and invasion of choroidal melanoma by targeting BRAF [13]. Bi et al. [14] have also revealed that lncRNA LINC01116 can promote colorectal cancer development by targeting miR-9-5p/STMN1. MiR-9 can inhibit epithelial OC cell proliferation via targeting the SDF-1/CXCR4 pathway [15]. And the overexpression of miR-9 promotes metastasis of serous OC by targeting E-cadherin [16].

Numerous studies showed that there was a significant correlation between miR-9 expression and the prognosis of various cancers, such as breast cancer [17], osteosarcoma [18], lung cancer [19], OC [20]. Nevertheless, the mechanism of miR-9 affecting OC is still unclear. Here, based on literature review and bioinformatic analysis of GEO datasets, we identified the possible molecular targets of OC and revealed the mechanism of miR-9 in OC.

## Materials and methods

### Selection of GEO datasets

To obtain gene chip profiles of OC, a search was performed in the Gene Expression Omnibus database (GEO, http://www.ncbi.nlm.nih.gov/geo/). The search strategy was as follows: (“ovarian cancer” OR “ovarian carcinoma” OR “ovarian tumor”) AND (“microRNA” OR “miRNA” OR “non-coding RNA” OR “small RNA”). The datasets related to miR-9 expression in OC tissues and normal ovarian tissues (NOTs) were included in our analysis.

The original data files were downloaded directly from the GEO database, and were preprocessed by R software (version 3.6.3). Based on the groups (OC group and normal ovarian group), the data in the original files were sorted into a Excel table. By using the gene probe file provided by the platform, the gene probes in the sorted Excel document were converted into the gene symbols. Finally, the gene expression data of miR-9 in OC and NOTs were screened.

### Literature retrieval

PubMed, Web of Science, Cochrane Library, and Embase databases were comprehensively searched for the literature on the correlation between miR-9 expression and prognosis of OC. The time of literature retrieval was up to November 2020. The search strategy was as follows: (“microRNA-9” OR “miRNA-9” OR “miR-9”) AND (“ovarian cancer” OR “ovarian carcinoma” OR “ovarian tumor”). Literature retrieval was performed independently by two investigators and finally cross-checked.

The studies published in English on the relationship between miR-9 expression and OC prognosis were included. While reviews, nonclinical studies, case reports, abstracts were excluded. The quality evaluation of the included studies was performed according to the Newcastle-Ottawa scale (NOS) [21]. Only those with 6 stars or above, which were considered to be of high quality, were included in this study.

### Gene ontology enrichment and target prediction analysis

Gene expression profiles of datasets GSE18520 and GSE36668 were obtained from GEO. The dataset GSE18520 consists of 53 OC samples and 10 NOTs samples, while GSE36668 consists of 4 OC samples and 4 NOTs samples. The data were analyzed on the GPL570 platform Affymetrix Human Genome U133 Plus 2.0 Array (Affymetrix; Thermo Fisher Science, Inc., Waltham, MA, USA).

To identify differentially expressed genes (DEGs) between OC tissues and NOTs, we employed the Limma package (version 3.6.3) in R/BioManager for processing. The adjusted *P*-value (adj. P.Value) was calculated using Benjamini-Hochberg method for controlling the false discovery rate (FDR), thus correcting false positives. Cut-off criterion was defined as *P* < 0.05 and |log2 (FC)| > 1. The platform annotation files downloaded from the database were adopted to convert the probe data in the matrix files into gene symbols.

The online websites miRWALK3.0 (http://zmf.umm.uni-heidelberg.de/apps/zmf/mirwalk2/miRretsys-self.html) and TargetScan (http://www.targetscan.org/vert_72/) were used to predict the target genes of hsa-miR-9-5p (TG-miR-9-5p). The overlapping genes between DEGs in OC and TG-miRNA-9-5p were revealed, and TG-miR-9-5p was also predicted using the online tool Venny 2.1.0 (https://bioinfogp.cnb.csic.es/tools/venny/). The gene overlaps were analyzed by Gene Ontology (GO) and visualized using the Bingo plugin of Cytoscape software (version3.7.2). Kyoto Encyclopedia of Genes and Genomes (KEGG) was analyzed by using DAVID database. FDR < 0.05 was considered as the cutoff criterion. In addition, the protein-protein interaction (PPI) network based on the gene overlaps was constructed by using STRING 11.0 (https://string-db.org/), and the interactions were visualized with CytoHubba plugin for Cytoscape software (version 3.7.2). Then the hub genes of PPI network were obtained. A confidence score of C ≥ 0.7 was set as the cutoff criterion.

### Survival analysis

The effect of the top 10 hub genes obtained in the PPI network on OC prognosis was assessed using Kaplan-Meier plotter (www.kmplot.com). The Kaplan-Meier plotter is capable to assess the effect of 54 k genes (mRNA, miRNA, protein) on survival in 21 cancer types, including breast (*n* = 6234), ovarian (*n* = 2190), lung (*n* = 3452), and gastric (*n* = 1440) cancers. Sources for the databases include GEO, European Genome-phenome Archive (EGA), and The Cancer Genome Atlas (TCGA). OC patients were divided into the high expression group and the low expression group based on the median expression of hub genes. The overall survival risk of OC was evaluated using Kaplan-Meier method, and the hazard ratio (HR) was used as a measure of effect.

### Statistical analysis

Stata 15.0 statistical software was used for data processing. MiR-9 expression levels in microarray datasets downloaded from GEO were expressed as mean ± standard deviation (SD). Independent two-sample t-test was used for analysis. Pooled analysis of miR-9 expression levels in different GEO datasets was performed using a random-effects model. If HR and its 95% CI of miR-9 expression and OC survival risk were provided in the included literature, they were directly adopted. If HR was not directly provided in the literature, but with survival curves, the data were then extracted using Engauge Digitizer software and then HR was calculated. Overall survival (OS) and progression-free survival (PFS) were used to evaluate the effect of miR-9 expression on OC prognosis. A fixed-effects model was used for the pooled analysis of these prognostic indicators. *P* < 0.05 was considered statistically significant.

## Results

### miR-9 expression in ovarian cancer based on GEO

**Fig. 1:**
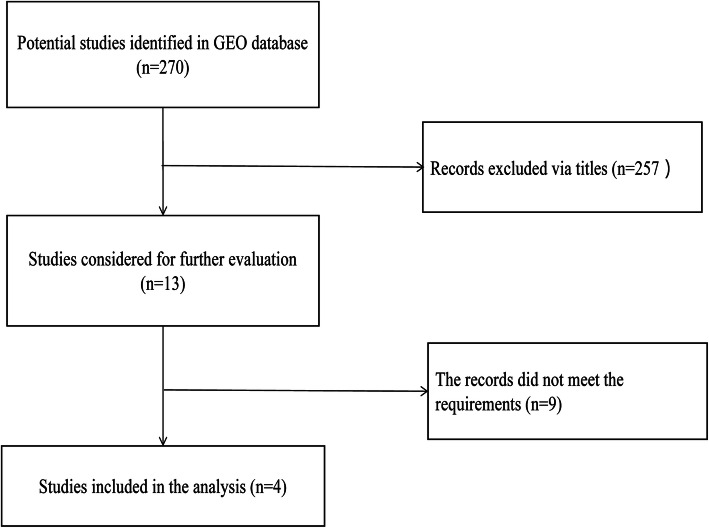
Research and screening flow diagram in GEO database. GEO: gene expression omnibus

**Fig. 2:**
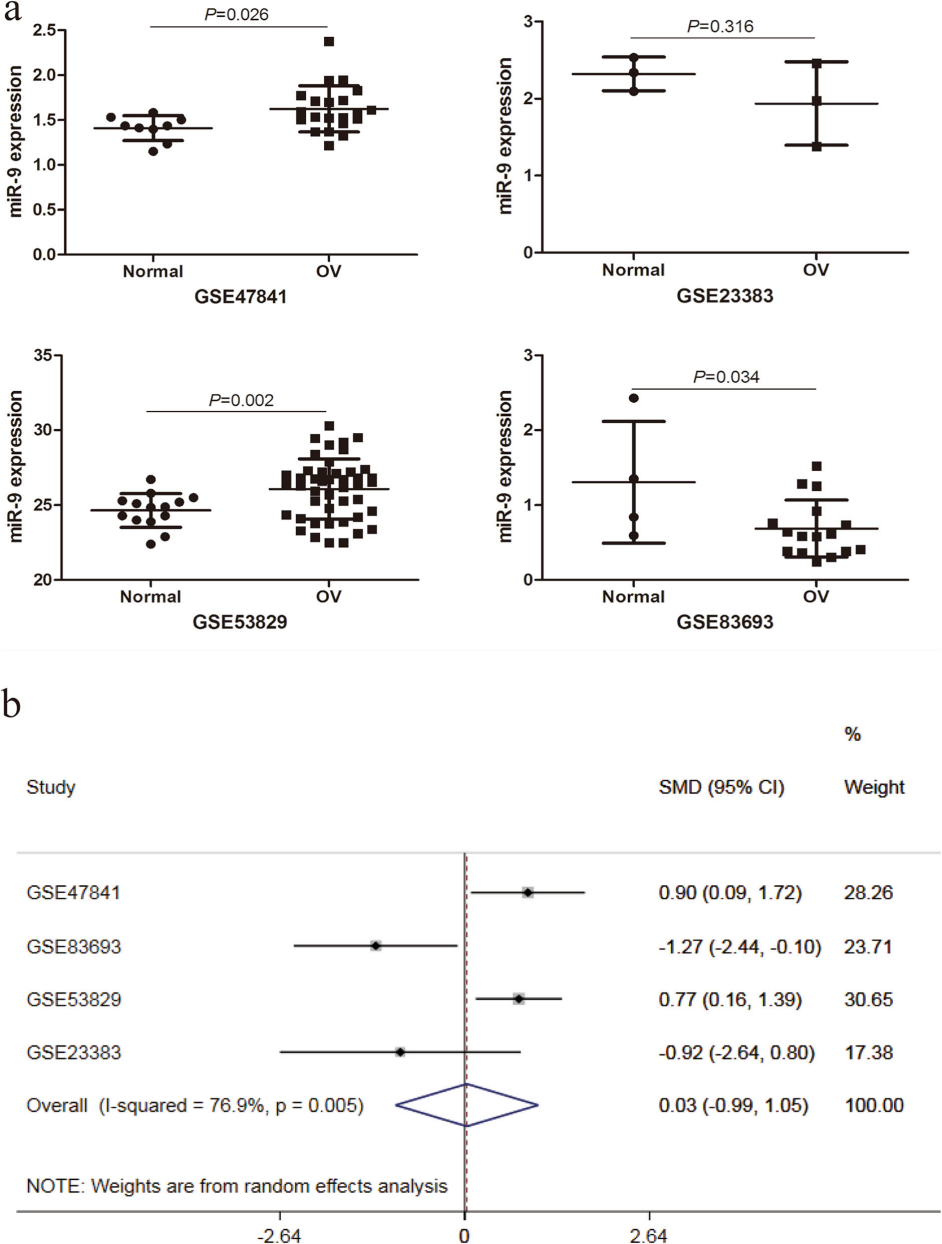
Expression of miR-9 in ovarian cancer and normal ovarian tissues in GEO. **a**:miR-9 expression in the datasets of GSE47841, GSE23383, GSE53829, GSE83693; **b**: Forest plot of differential expression of miR-9-5p in the GEO datasets. GEO: gene expression omnibus

The screening process of GEO datasets is shown in Fig. 1. A total of 4 GEO datasets (GSE47841, GSE83693, GSE53829, and GSE23338) were collected to assess miR-9 expression in OC tissues and NOTs. The miR-9 expression in OC tissues was significantly higher than that in NOTs in the datasets GSE47841 and GSE53829, while it was significantly lower than that in NOTs in the dataset GSE83693 (all *P* < 0.05). And no significant difference was identified in the GSE23338 dataset. The above comparison results are shown in Table 1 and Fig. 2a. However, the results of the random-effects model showed no significant difference in miR-9 expression between the OC tissues and NOTs (SMD = 0.03, 95% CI: − 0.99-1.05, *P* = 0.952). The forest plot is shown in Fig. 2b.

**Table 1:** Basic characteristics of miR-30a related Research on Ovarian Cancer in GEO dataset

Study	Ovarian cancer tissue			Normal ovarian tissue			t	*p*
	Mean	SD	n	Mean	SD	n		
GSE47841	1.62	0.26	21	1.41	0.14	9	2.344	0.026
GSE83693	0.69	0.38	16	1.3	0.81	4	2.291	0.034
GSE53829	26.08	2.01	45	24.65	1.13	14	3.344	0.002
GSE23383	1.94	0.54	3	2.32	0.22	3	1.146	0.316
Total	SMD (95%CI) = 0.03(−0.99 ~ 1.05), *p* = 0.952;I^2^ = 76.9%, *p* = 0.005							

*GEO* Gene expression omnibus, *SD* Standard difference, *SMD* Standard mean difference

### Effect of miR-9 expression on the prognosis of ovarian cancer

**Fig. 3:**
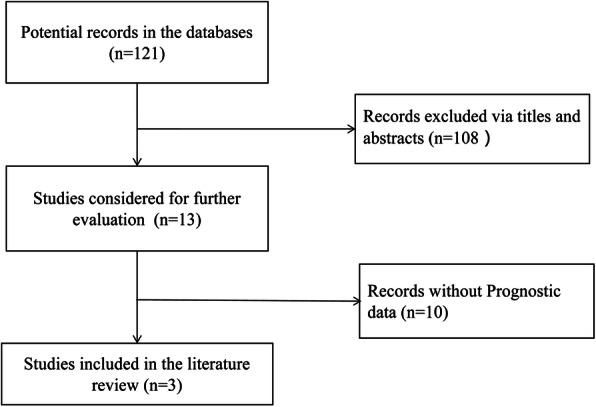
Flow diagram for screening of studies on the relationship between miR-9 expression with the prognosis of patients with ovarian cancer

**Fig. 4:**
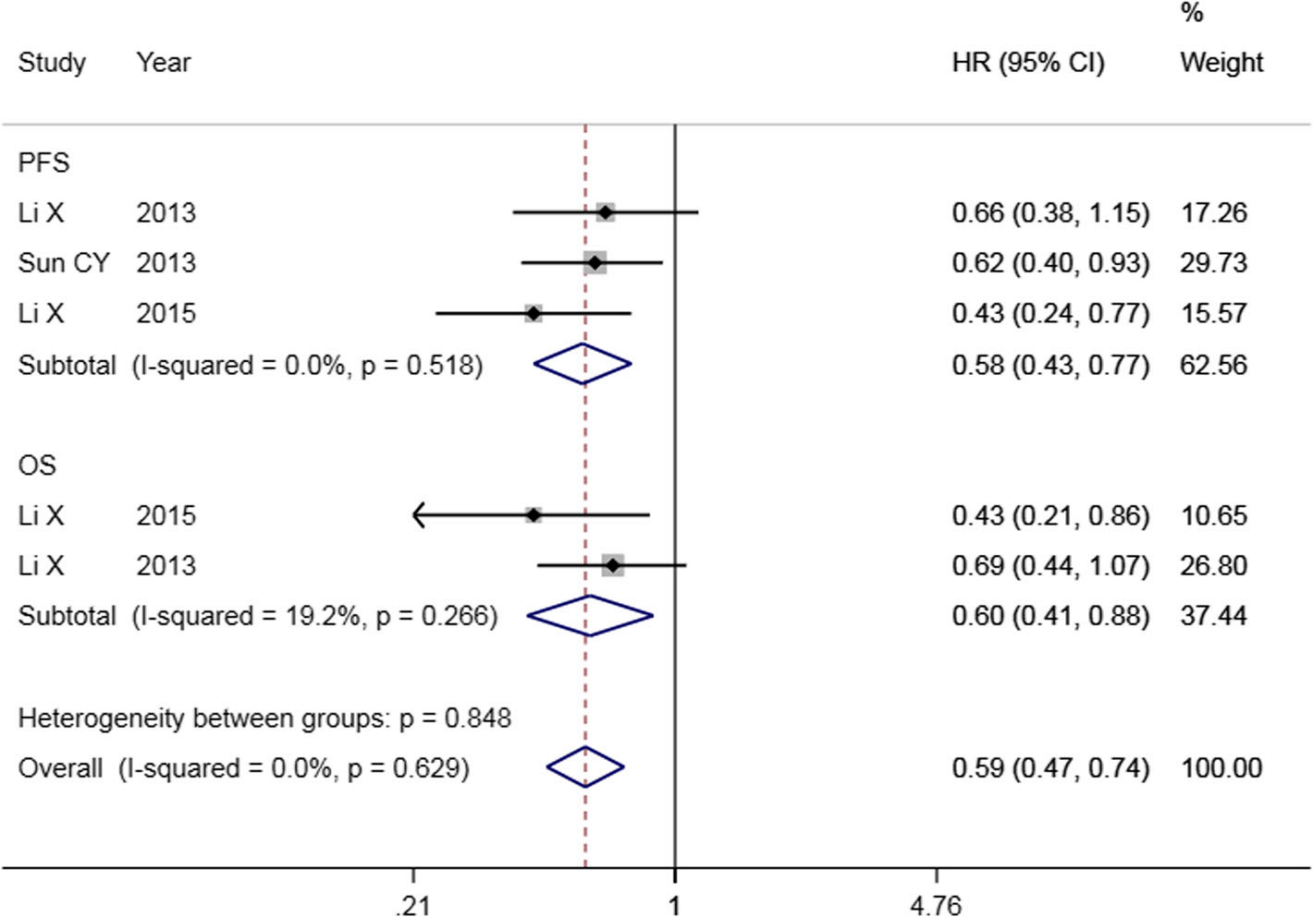
Forest plot of the correlation between miR-9 expression and prognosis in patients with ovarian cancer. OS:Overall survival; PFS: progression-free survival

The screening process of the literature is shown in Fig. 3. A total of 3 studies with NOS scores of more than 6 stars that met the inclusion criteria were identified [20, 22, 23]. Among them, 2 studies contained OS data, and 3 studies contained PFS data. The results of the fixed-effect model (Fig. 4) showed that high expression of miR-9 was beneficial for the survival of OC patients (OS: HR = 0.60, 95% CI: 0.41–0.88; PFS: HR = 0.58, 95% CI: 0.43–0.77). The differences were statistically significant.

### miR-9 target gene prediction and DEGs screening

**Fig. 5:**
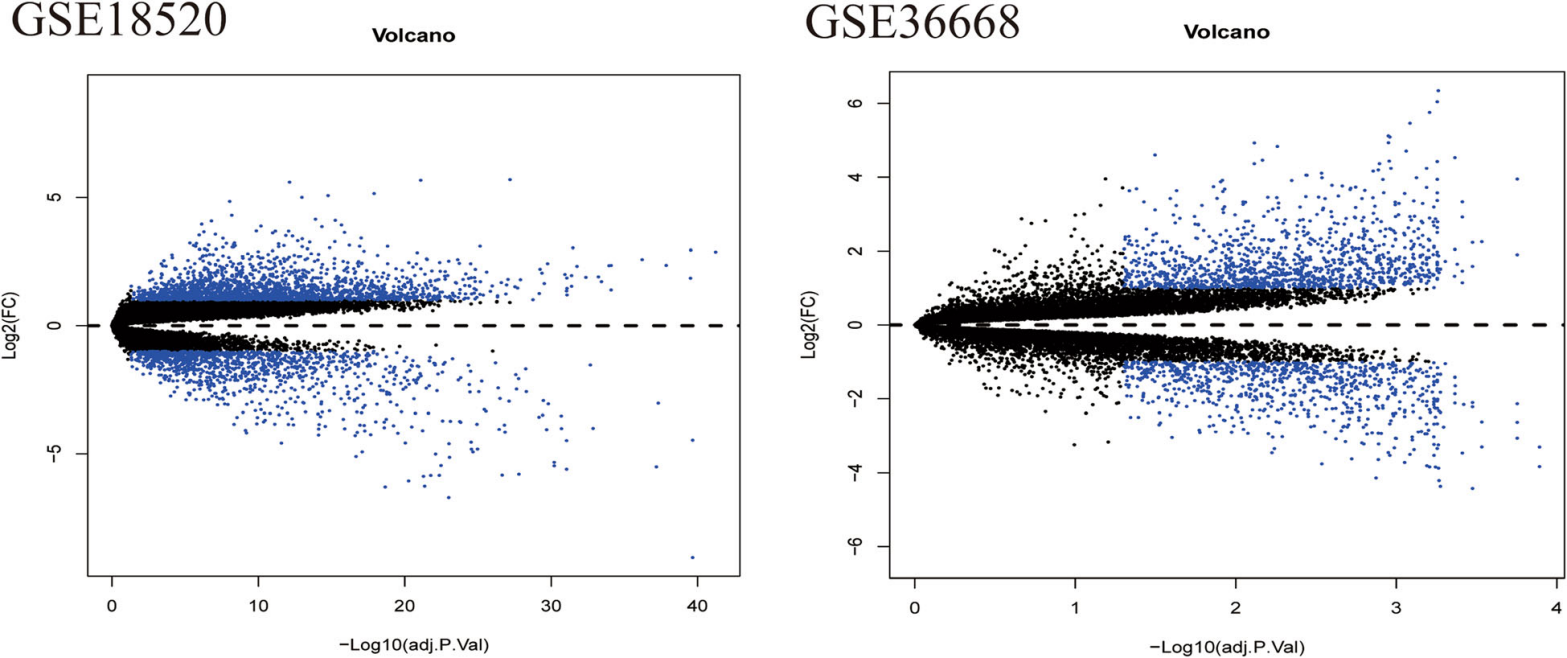
Volcano plot of Genome-wide mRNA detected by two ovarian cancer-related datasets from GEO. Aberrantly expressed mRNAs with *P* < 0.05 and |log (FC)| > 1 were represented by blue. up-regulated genes were indicated by blue plots above, down-regulated genes were indicated by blue plots below, and normally expressed mRNAs were indicated by black plots . The y-axis shows the fold-change value between the mRNAs expression in ovarian cancer and normal ovarian tissues. The x-axis represents -log10 of adj. *P*-Value. adj. *P*-Value, adjusted *P* value; FC, fold change; GEO, gene expression omnibus

**Fig. 6:**
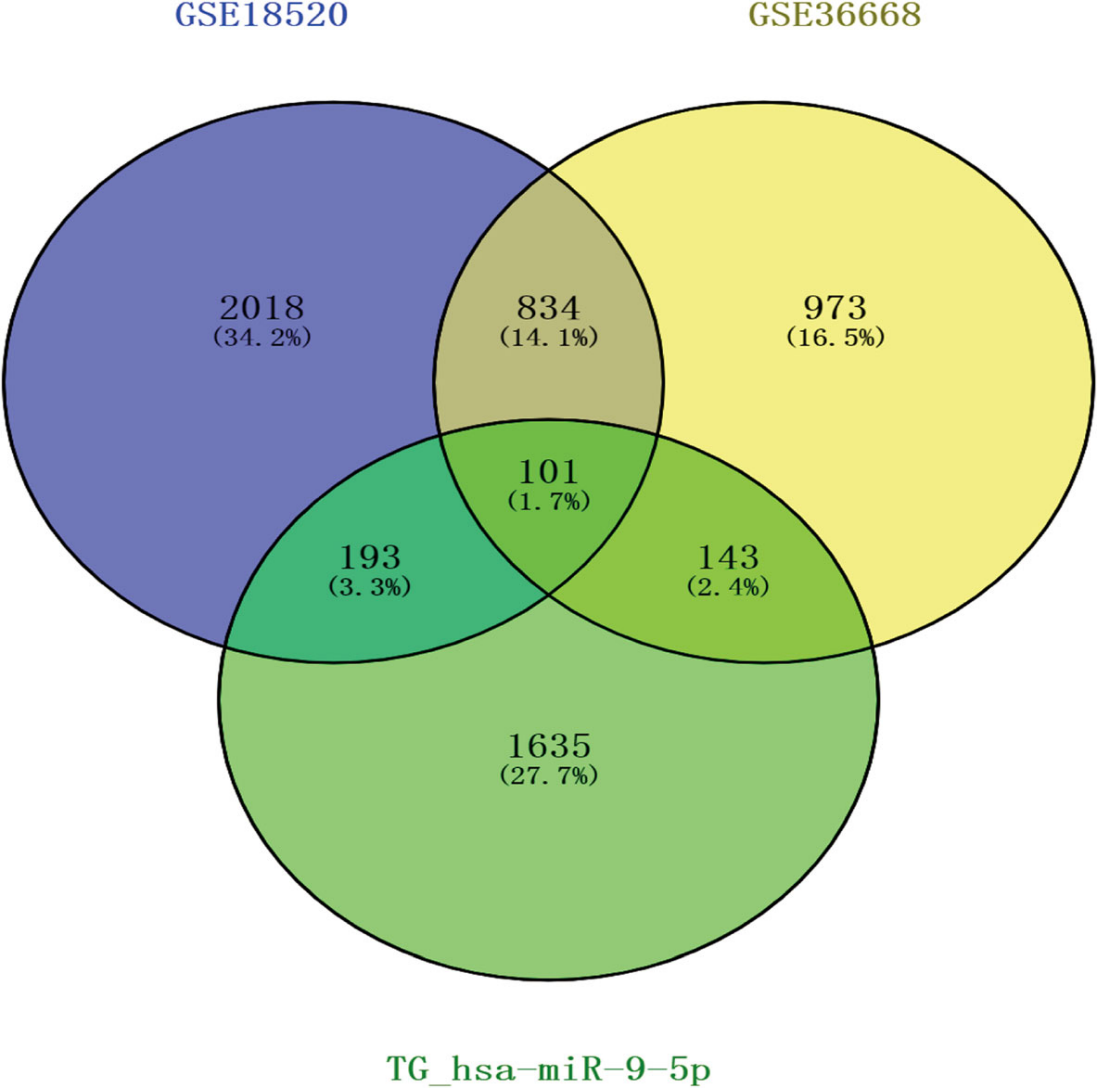
The Venn map of hsa-miR-9-5p related DEGs in TG_miR-9-5p and other two GEO datasets, and the overlapping region represents the recognized DEGs. DEGs: differentially expressed genes; TG_miR-9-5p, target genes of hsa-miR-9-5p

In the GSE18520 and GSE36668 datasets, 3146 and 2051 DEGs were identified, respectively. Volcano plots of gene expression differences in these two datasets are shown in Fig. 5. A total of 935 common DEGs were selected in these two datasets (Fig. 6) by using a Venn diagram. Then, based on miRWALK3.0 and TargetScan, 2072 TG-miR-9-5p were predicted, 101 of which were validated in the 935 common DEGs (Fig. 6). Among these genes associated with hsa-miR-9-5p, 50 were up-regulated and 51 were down-regulated in OC tissues compared with NOTs. The top 10 up-regulated and down-regulated hsa-miR-9-5p-related DEGs in the dataset GSE18520 are listed in Table 2. Among them, genes such as MUM1L1, CALB2, and VGLL3, were important has-miR-9-5p-related target genes of OC.

**Table 2:** differentially expressed genes of hsa- miR-9-5p in ovarian cancer tissues and normal ovarian tissues in GSE18520 dataset (Top 10)

DEGs	logFC	P.Value	adj. P.Val
down-regulated genes			
MUM1L1	−6.262299987	4.62E-24	4.32E-22
CALB2	−5.507761536	2.48E-41	6.45E-38
VGLL3	−4.030605297	4.30E-16	7.52E-15
RNF128	−3.930363883	1.39E-16	2.65E-15
ANKRD29	−3.879855847	4.43E-30	1.54E-27
SYT4	−3.848423623	4.75E-28	1.16E-25
PEG3	−3.754622588	5.71E-12	4.33E-11
ADH1B	−3.653994292	3.68E-21	1.81E-19
EFEMP1	−3.428021105	1.89E-12	1.56E-11
ADAMTS3	−3.417352741	4.99E-12	3.83E-11
Up-regulated genes			
MCM10	2.742105511	1.65E-20	7.07E-19
CTCFL	3.123690136	8.30E-07	2.61E-06
INHBB	3.385619965	7.36E-15	9.97E-14
FOXQ1	5.001861129	7.78E-15	1.05E-13
NCAPG2	1.251323035	2.01E-08	8.26E-08
LYZL1	2.351482953	4.01E-38	7.98E-35
C2orf88	2.365573525	2.01E-08	8.26E-08
LZTS3	2.419404533	2.90E-21	1.46E-19
EHF	2.429320583	2.95E-17	6.47E-16
PLAG1	2.408785627	5.21E-08	2.00E-07

*DEG* Differentially expressed gene, *FC* Fold change

### GO and KEGG analysis of miR-9-related DEGs in ovarian cancer

**Fig. 7:**
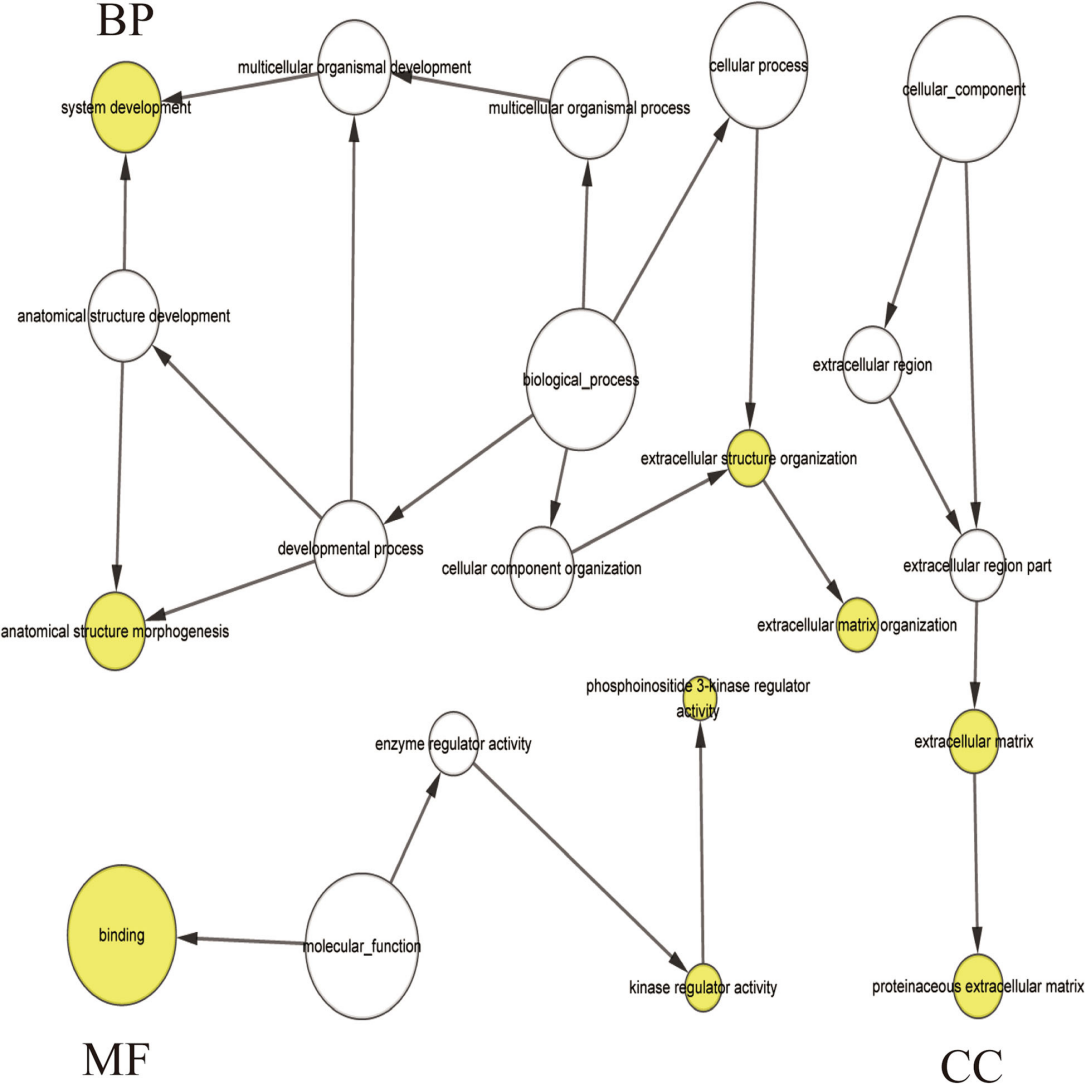
Results of GO enrichment Analysis of the recognized DEGs by using the Bingo plugin of Cytoscape software. The yellow circle represents functional enrichment, and the larger the circle, the darker the color, the more genes are enriched in this pathway. The connecting lines represent the association between gene and gene. GO: Gene Ontology.DEGs: differentially expressed genes

As shown in Table 3, the partial results of GO and KEGG enrichment analyses revealed that a number of miR-9-related target genes were involved in biological processes such as developmental process, anatomical structure development, extracellular matrix structural constituent, cell junction, and axon guidance. The results of GO enrichment analysis were visualized using the Bingo plugin for Cytoscape software (Fig. 7).

**Table 3:** Functional and pathway enrichment analysis of hsa-miR-9-5p related differentially expressed genes in Ovarian Cancer

Term	Description	Count	FDR
Biological processes			
GO:0032502	developmental process	46	0.0172
GO:0048856	anatomical structure development	45	0.0316
GO:0071840	cellular component organization or biogenesis	45	0.0199
GO:0016043	cellular component organization	44	0.0266
GO:0007275	multicellular organism development	42	0.0183
Molecular Function			
GO:0005201	extracellular matrix structural constituent	5	0.0202
Cellular Component			
GO:0030054	cell junction	16	0.0181
KEGG pathway			
Hsa-1,474,244	Extracellular matrix organization	8	0.0417
Hsa-422,475	Axon guidance	11	0.0459

*FDR* False discovery rate, *Count* The number of enriched genes in each term, *GO* Gene ontology, *KEGG* Kyoto Encyclopedia of Genes and Genomes

### PPI network construction and hub gene selection

**Fig. 8:**
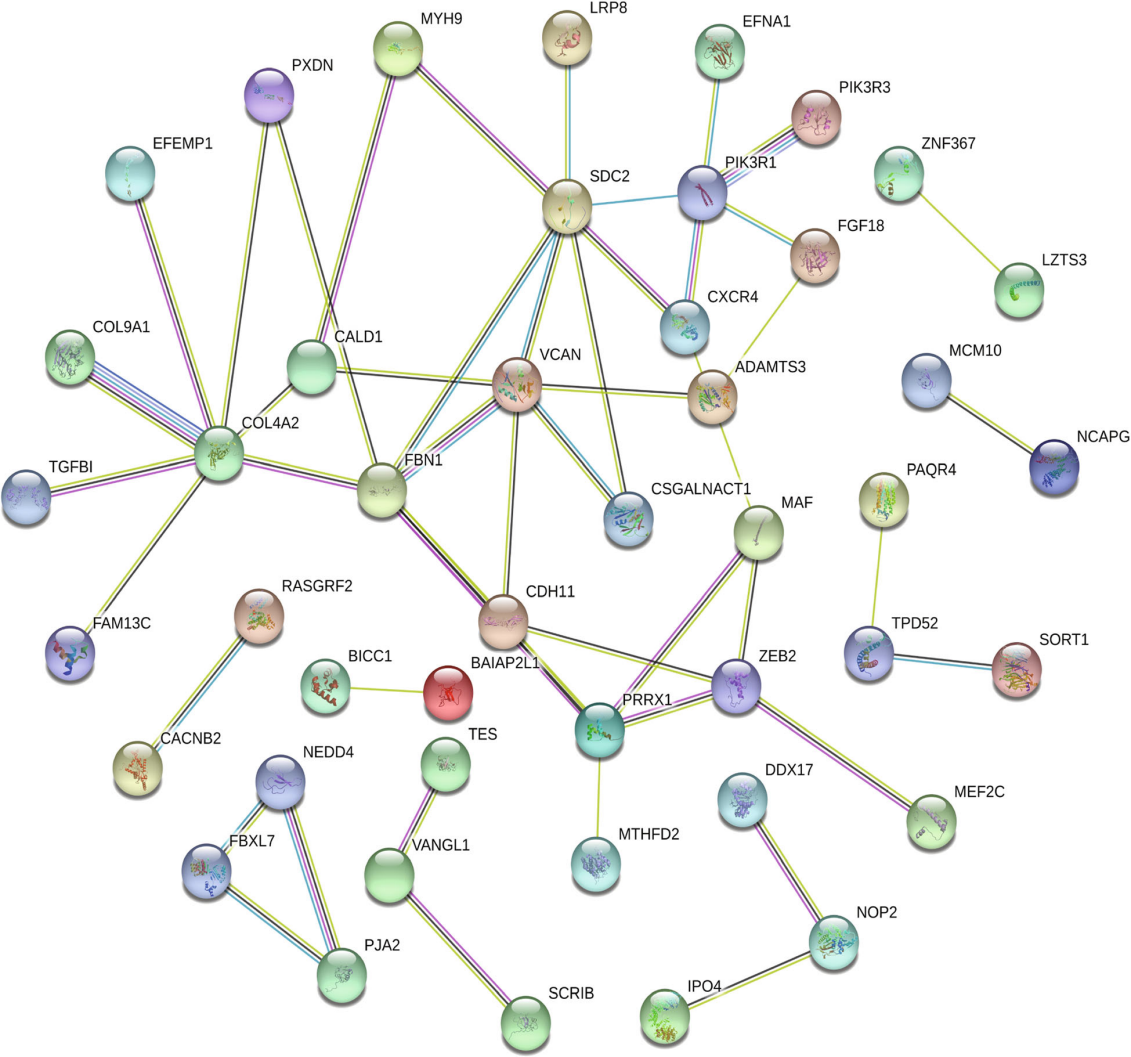
Protein-protein interaction network of hsa-miR-9-5p-related DEGs. The lines represent interaction relationship between nodes. DEGs, differentially expressed genes. The line between the circle nodes represents the interaction between the two proteins linked by the line. Colored nodes:query proteins and first shell of interactors;white nodes:second shell of interactors;empty nodes:proteins of unknown 3D structure;filled nodes:some 3D structure is known or predicted..DEGs: differentially expressed genes

**Fig. 9:**
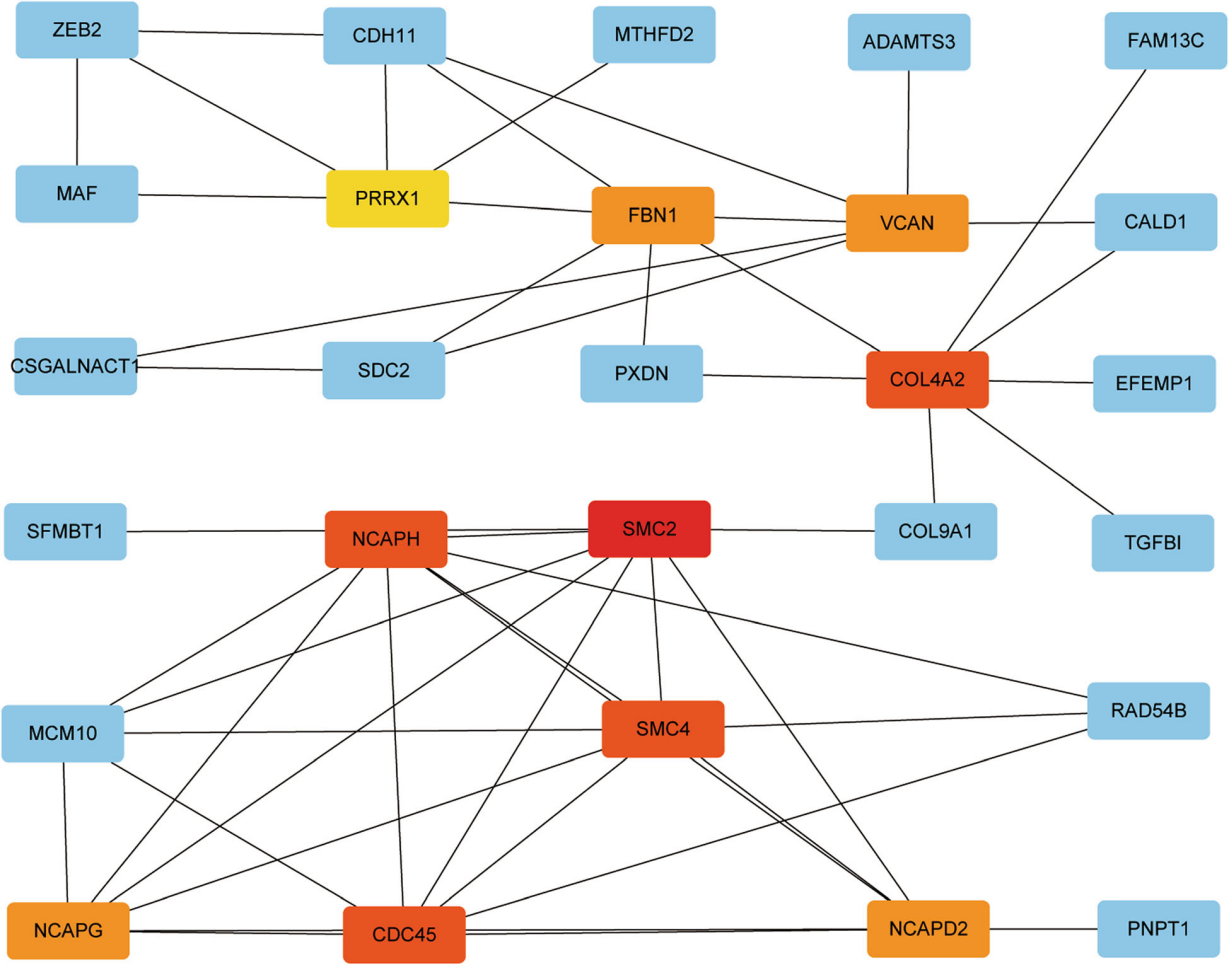
Protein-protein interaction network. (A): Protein-protein interaction network of hube genes of hsa-miR-9-5p-related DEGs. The red and yellow represent the top 10 Hub genes. The darker the color, the stronger the association with other genes in the PPI network. The lines represent interaction relationship between nodes. DEGs:differentially expressed genes

The PPI network of miR-9-related DEGs was constructed with 101 nodes and 48 edges, which included 50 up-regulated genes and 51 down-regulated genes (Fig. 8). By using the degree as the ranking criterion, the top 10 genes were selected as hub genes (Fig. 9). Thus, there was a close relationship among hub genes.

**Fig. 10:**
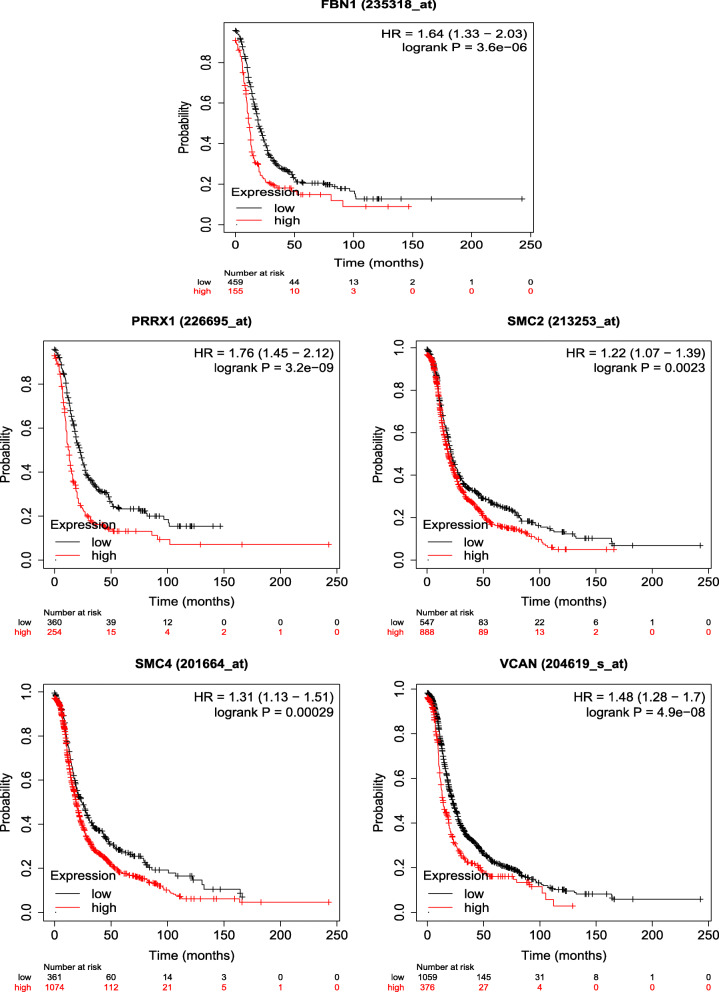
Association between the expressions of FBN1, PRRX1, SMC2, SMC4, and VCAN and the prognosis of patients with ovarian cancer. According to the median expression level, patients with ovarian cancer were divided into high expression group and low expression group. HR:hazard ratio

### Survival analysis

The prognostic values of the top 10 hub genes selected from the PPI network were evaluated using Kaplan-Meier plotter. The results showed that 5 of the top 10 hub genes were significantly associated with the risk of survival in OC patients. High expression of these 5 hub genes led to a decrease of survival rate in OC patients (Fig. 10), namely FBN1 (HR = 1.64, *P* < 0.05), PRRX1 (HR = 1.76, *P* < 0.05), SMC2 (HR = 1.22, *P* < 0.05), SMC4 (HR = 1.31, *P* < 0.05), and VCAN (HR = 1.48, *P* < 0.05). The differences were statistically significant.

## Discussion

In this study, the GEO datasets related to expression of miRNAs in OC were systematically searched. Based on the comparison of expression profiles of miRNAs in OC tissues and NOTs, the abnormal expression of miR-9 associated with OC was identified. Based on the comprehensive literature retrieval, it was found that high expression of miR-9 was favorable for OC patients, which could be confirmed by both OS and PFS data. Although the number of included studies is relatively small, this conclusion is still of great significance. In addition, GO, KEGG, PPI network, and survival analysis were adopted to identify and analyze the novel makers and potential targets of miR-9 involved in the regulation of essential biological processes in OC.

To date, few studies have investigated miR-9 characteristics in OC, and there is a lack of data on the miR-9 expression in literature. Therefore, we analyzed the expression of miR-9 in 4 microarray datasets from GEO. The results showed that miR-9 expression was significantly up-regulated in 2 datasets and significantly down-regulated in 1 dataset, while no significant difference in 1 dataset. However, the pooled analysis of miR-9 expression in these 4 datasets revealed no significant difference. Thus it is still inconclusive about the expression of miR-9 in OC. The reason for the above inconsistency may be related to the histological types of OC. The survival analysis pointed out a positive relationship between high expression of miR-9 and OC prognosis. But the specific mechanism is still unknown. In the study of Sun et al. [23], miR-9 mediates the down-regulation of BRCA1 gene, which in turn hinders the repair of DNA damage in OC, suggesting that the therapeutic effect of OC can be improved by increasing the sensitivity of cancer cells to DNA damage. Li et al. [20] have proved that miR-9 may have affected paclitaxel chemosensitivity in OC patients by targeting CCNG1.

MiR-9 is one of the most critical miRNAs in the regulation of OC. Zhang et al. [24] have reported that CircPLEKHM3 exerts tumor-suppressive effects in OC cells by targeting the miR-9/BRCA1/DNAJB6/KLF4/AKT1 axis and can be used as a prognostic indicator and therapeutic target for OC. MiR-9 has been demonstrated to promote the epithelial-to-mesenchymal transition of OC cells by inhibiting E-cadherin [25]. Moreover, epigenetic modification of miR-9 is involved in the pathogenesis and progression of OC, and it has a certain diagnostic value for OC [26].

In the current study, we found that novel candidate target genes of miR-9 are involved in the regulation of vital biological processes in OC, such as Calbindin2 (CALB2), Vestigial like family member3 (VGLL3). CALB2 is a 29-kDa calcium-binding protein of the EF-Hand family, which is a family of proteins containing calcium-binding motifs composed of two helices (E and F) [27]. CALB2 has been shown to regulate colorectal cancer patient sensitivity to 5-fluorouracil by modulating the intrinsic apoptosis pathway [28]. But there are few studies on the role of CALB2 in OC. VGLL3, as a member of vestigial like family of proteins, is associated with epithelial OC [29] and soft tissue sarcoma [30].

Our study also found 5 hub genes with a significant correlation with OC prognosis, such as FBN1, PRRX1, and SMC4. Their high expression contributes to the poor prognosis of OC patients. FBN1 is the coding gene for fibrillin-1 and the main component of extracellular matrix microfibers, which is crucial in maintaining the morphological integrity and normal function of connective tissue [31]. Studies have reported that high expression of FBN1 decreases the OS of serous ovarian cancer [32], and it increases the risk of lymph node metastasis [33]. PRRX1 has been demonstrated as a novel inducer of the epithelial-mesenchymal transition of tumor cells, such as breast cancer [34], colorectal cancer [35], and OC [36]. SMC4 protein is associated with tumor development, and the main biological function of SMC4 protein is the involvement in the dynamic changes of higher-order chromosomal structures, such as chromosome condensation and separation, DNA recombination, and repair of DNA damage [37, 38]. These hub genes are expected to be new predictors of OC prognosis, and become new targets for OC treatment.

The findings of this study have great and far-reaching significance for the clinical targeted therapy of OC patients. It is a long-term and arduous task for medical workers to explore the mechanism of miRNA in OC, to find effective targets, to develop effective targeted drugs for targeted therapy, so as to prolong the survival time of OC patients and improve their quality of life.

## Conclusion

In summary, miR-9 is critical in the biological processes of OC. However, further in vitro and in vivo experiments are still required for its pathogenesis, thus validating the role of miR-9-regulated molecular networks in OC.

## Data Availability

The datasets used and/or analysed during the current study are available from the corresponding author on reasonable request.

## Abbreviations

miRNAs: MicroRNAs
OC: Ovarian cancer
miR-9: hsa-miR-9-5p
GEO: Gene Expression Omnibus
GO: Gene Ontology
KEGG: Kyoto Encyclopedia of Genes and Genomes
PPI: Protein-protein interaction
ACS: American Cancer Society
NOTs: Normal ovarian tissues
FDR: False discovery rate
SD: Standard deviation
OS: Overall survival
PFS: Progression-free survival
HR: Hazard ratio
